# Exploring fatigue in clinically depressed adolescents: a secondary analysis of the IMPACT study sample

**DOI:** 10.1186/s40359-026-04942-3

**Published:** 2026-06-12

**Authors:** Nina Higson-Sweeney, Barnaby D. Dunn, Noah Marshall, Kate Cooper, Shirley Reynolds, Ian M. Goodyer, Maria E. Loades

**Affiliations:** 1 Department of Psychology, University of Bath, Claverton Down, Bath, BA2 7AY UK; 2 Present Address: Department of Experimental Psychology, University of Oxford, Life and Mind Building, South Parks Road, Oxford, OX1 3EL UK; 3 Mood Disorders Centre, University of Exeter, Exeter, EX4 4QG UK; 4 Department for Health, University of Bath, Claverton Down, Bath, BA2 7AY UK; 5 Department of Clinical, Educational and Health Psychology, University College London, Torrington Place, London, WC1E 7HB UK; 6 School of Psychology and Clinical Language Sciences, University of Reading, Earley, Reading, RG6 6ET UK; 7 Department of Psychiatry, University of Cambridge, Robinson Way, Cambridge, CB2 0SZ UK

**Keywords:** Adolescence, Depression, Fatigue, Psychological interventions, Psychosocial functioning, Secondary analysis

## Abstract

**Background:**

Fatigue is a highly disabling symptom of adolescent depression, yet little is known about prevalence, associated characteristics, nor how fatigue might change during psychological intervention.

**Methods:**

An exploratory secondary analysis was conducted using trial data from 465 adolescents (aged 11–17 years old) diagnosed with major depressive disorder who were randomised to receive one of three psychological interventions. Fatigue was measured as a binary variable (fatigue status; fatigued versus non-fatigued). We explored the prevalence of fatigue, associated demographic and clinical characteristics, changes in fatigue following psychological intervention, and the impact of baseline fatigue on improvements in depression severity and psychosocial functioning.

**Results:**

Clinically significant fatigue was self-reported by 73% of adolescents at baseline. From self-report and diagnostic interviews, fatigue status was consistently associated with older age and higher depression severity. Through self-report only, depressed adolescents were also more likely to be fatigued if they were an ethnic minority, had more comorbidities, and higher psychosocial impairment. Fatigue status significantly decreased across all timepoints, irrespective of treatment arm, and continued to decrease after treatment was terminated. Furthermore, fatigue did not significantly predict overall treatment response or differential response between arms in relation to depression severity and psychosocial functioning. However, up to a third of depressed adolescents still reported residual fatigue at 86-weeks follow-up.

**Conclusions:**

In this exploratory study, fatigue is commonly reported by depressed adolescents and consistently associated with older age and higher depression severity, which are characteristics that could aid identification. Fatigue may decrease when receiving evidence-based psychological interventions for adolescent depression, but a proportion are left with residual symptoms. Further research is warranted to expand on these tentative findings.

**Supplementary Information:**

The online version contains supplementary material available at 10.1186/s40359-026-04942-3.

## Introduction

Fatigue is common in adolescence, with reports that as many as one in three adolescents experience it [1]. Defined as the subjective experience of extreme physical and/or mental exhaustion following normal activities [2], prolonged fatigue has been associated with poor outcomes for adolescents, including school absenteeism [3], reduced engagement with leisure activities, impaired social relationships [4] and subsequent mental health problems in adulthood [5]. As adolescence is a period of rapid biological, cognitive, social, and emotional change [6], fatigue has the potential to detrimentally impact typical developmental trajectories, making it a public mental health concern.

Fatigue is a symptom of major depressive disorder (MDD) [7] that is reported by 43% to 97% of adolescents with a diagnosis of MDD [8–10]. This wide spread of estimates may reflect differences between instruments utilised to measure fatigue (e.g., self-report versus diagnostic interview), yet no research has directly compared alignments between different fatigue measures, despite potential clinical implications for accuracy and triangulation during assessment.

A growing number of studies have identified that fatigue and depression in adolescents independently correlate and covary over time [11] and are predictive of one another [12–14]. A longitudinal study by Tham et al. [15] also identified that, in comparison to healthy adolescents and adolescents with chronic pain, depressed adolescents experience more severe fatigue. Additionally, recent network analyses including adolescent samples have identified fatigue as the most commonly reported symptom of depression with the highest strength centrality (i.e., the symptom with the strongest connections to other depressive symptoms [16]), and as the symptom with the highest outstrength centrality (i.e., strongest ability to predict other symptoms over time [17]). This suggests that fatigue may play an integral role in influencing other depressive symptoms and could be a maintaining factor of adolescent MDD if left untreated. However, despite increasing recognition of the clinical significance of fatigue within adolescent depression, it is often not assessed and/or targeted within routine child and adolescent mental health services (CAMHS) provision for MDD [18]. This could be due to a lack of understanding or health literacy around fatigue, combined with a lack of knowledge regarding indicative factors of fatigue in adolescent depression specifically. As little research has been conducted in this area, it is not currently known what demographic or clinical characteristics may be associated with fatigue in this population, which could aid early identification prior to treatment.

The potential detrimental consequences of unaddressed fatigue can be seen in adult populations, with 20% to 38% of remitted depression patients reporting substantial fatigue as a residual symptom [19–21]. This is problematic, as residual symptoms of depression are predictive of greater risk of subsequent relapse and ongoing functional and psychological impairment [22, 23]. Untreated fatigue in adolescents with MDD may lead to similar consequences, but this has yet to be comprehensively explored. Due to important developmental and contextual differences, we cannot assume that findings from adults will generalise to adolescents [24, 25]. They do, however, still indicate cause for concern.

In the UK, guidelines from the National Institute for Health and Care Excellence [26] recommend that adolescents with moderate to severe MDD are referred to CAMHS for treatments like cognitive behavioural therapy (CBT) with or without antidepressants. Whilst systematic reviews and meta-analyses of randomised controlled trials (RCTs) have reached conclusions that are generally in favour of these treatment options [27–29], effects of treatments are usually moderate at best ([27]: Hedges’ *g* = 0.21 to 0.36; [28]: standardised mean difference [SMD] = -0.41 to -0.20; [29]: SMD = -1.37 to -0.16). The studies themselves also often have significant issues regarding heterogeneity and methodological quality [30]. This suggests that whilst these psychological interventions work for some adolescents, they do not work for all. This could be because not all symptoms of MDD are adequately addressed during treatment and could interfere with treatment efficacy. It is unknown whether fatigue in adolescent MDD is such a symptom, although indications from the adult literature, combined with qualitative findings regarding lack of assessment in CAMHS [18] and the impact of fatigue-induced psychosocial impairment on treatment engagement [31, 32], suggest that this could be the case. It is therefore pertinent to explore whether fatigue responds to or interferes with psychological treatments for depression.

As far as we are aware, no studies to date have been conducted investigating the effectiveness of psychological interventions for adolescent MDD in addressing fatigue. An efficient way to address this is to conduct exploratory secondary analyses on existing high-quality depression treatment trials that incorporate measures of fatigue. As scales pertaining to fatigue in depression have yet to be validated in adolescents (e.g., the Fatigue Associated with Depression Questionnaire [33]), and trials on adolescent depression do not tend to incorporate specific measures of fatigue as it is not a primary or secondary outcome, it is necessary to extract fatigue items embedded within global depression scales. Whilst such an approach is suboptimal, given limitations like lower reliability, range restriction, and insufficiency in capturing multidimensional constructs, it can be a useful starting point and appropriate method in the context of exploratory research [34].

We used data from the Improving Mood with Psychoanalytic and Cognitive Therapies (IMPACT) trial, which was a single-blind pragmatic superiority RCT comparing the clinical and cost effectiveness of three psychological treatments for adolescent MDD [8, 35]. This trial utilised the Kiddie Schedule for Affective Disorders and Schizophrenia for School-Age Children (K-SADS) [36] and the Mood and Feelings Questionnaire (MFQ) [37], both of which include a fatigue item. In the main trial outcomes paper, 73% of the sample were identified as having clinically significant levels of fatigue based on the K-SADS diagnostic interview [8]. However, the prevalence of self-reported fatigue on the MFQ has not previously been reported. It is feasible that there could be differences between the two [38], with potential implications for accurate assessment and treatment.

Subsequently, the current study conducted exploratory post-hoc analyses on IMPACT data to address the following research questions:

1. What is the prevalence of fatigue at baseline in self-report and diagnostic interview terms, and do these align?
2. What clinical and demographic characteristics of depressed adolescents are associated with fatigue?
3. What impact does treatment have on fatigue levels?
4. Does fatigue at baseline predict response (depression improvement) to acute (36-week) treatment, and does this vary as a function of treatment arm?
5. Does fatigue at baseline predict response (psychosocial functioning improvement) to acute (36-week) treatment, and does this vary as a function of treatment arm?

## Methods

### Participants and recruitment

Participants were adolescents aged 11–17 years who met the criteria for current MDD and were recruited from 15 National Health Service (NHS) CAMHS clinics across three regions in England. 470 adolescents were recruited and randomised (1:1:1 ratio) to one of three treatment arms: CBT, short-term psychoanalytic psychotherapy (STPP), and a brief psychosocial intervention (BPI), which was the active reference condition. Each treatment was delivered in routine CAMHS by clinicians following pre-specified treatment manuals, which varied in terms of (per protocol) duration and number of sessions (CBT: ≤ 20 sessions across 30 weeks; STPP: ≤ 28 sessions across 30 weeks; BPI: ≤ 12 sessions across 20 weeks). Five participants withdrew post-randomisation but prior to the beginning of treatment and requested for their data to be deleted. The remaining 465 participants had an average age of 15.61 years (*SD* = 1.42; range = 11.30-17.99), with 74.8% identifying as female, and 82.2% identifying as white. 154 participants received CBT, 156 received STPP, and 155 received BPI, with no clear demographic differences between arms.

### Ethics

The IMPACT study was approved by the Cambridgeshire 2 Research Ethics Committee (09/H0308/137). The current secondary analysis was approved by the University of Bath Psychology Research Ethics Committee (21–201). We pre-registered our data analysis plan on the Open Science Framework (OSF) prior to accessing data: 10.17605/OSF.IO/EV2UP.

### Measures and procedure

Three measures were used in the current study, across three timepoints: baseline (prior to randomisation), post-treatment (36-weeks post-randomisation), and follow-up (86-weeks post-randomisation). Age, sex, ethnicity, and medication status at baseline were also measured. Further information regarding measures and procedure can be found in Goodyer et al. [35].

#### Diagnostic interview

The K-SADS [36] is a semi-structured diagnostic interview designed to establish the presence of DSM-IV diagnoses in children and adolescents. Each symptom is rated by a trained interviewer on a 3-point scale, with ratings of 3 (“threshold”) considered clinically significant. For diagnoses, the K-SADS has good interrater reliability (98%, range = 93%-100%) and excellent to good test-retest reliability (κ = 0.63-0.90; [36]).

For the purposes of the current study, the individual item ‘Fatigue, Lack of Energy and Tiredness’ within the K-SADS Depression Supplement was used as one of the primary measures of fatigue, recoded into a binary categorical variable of ‘fatigue status’ (fatigued versus non-fatigued; 1, 2 = ‘non-fatigued’; 3 = ‘fatigued’). This item is accompanied by a list of prompts which interviewers can flexibly use to aid their judgement on the presence of fatigue (e.g., ‘Do you feel like this all the time?’, ‘Do you take naps because you feel tired?’).

The K-SADS was also used to measure the presence or absence of comorbid diagnoses. A continuous comorbidity variable was created from the sum total of concurrent comorbid diagnoses (e.g., generalised anxiety disorder, obsessive-compulsive disorder, conduct disorder), resulting in a variable ranging from 0 (no comorbid diagnoses) to 11 (11 comorbid diagnoses).

#### Self-report measures

The MFQ Child Self-Report [37] is a 33-item measure which asks adolescents to consider each item in relation to how they have felt over the past 2 weeks. The measure was designed to cover symptom areas specified in the DSM-IV for an episode of MDD. All items are scored on a 3-point Likert scale ranging from 0 (“not true”) to 2 (“true”), giving a range of 0–66, with higher scores representing greater likelihood of increased number and severity of depression symptoms. The MFQ is well-validated, with robust psychometric properties [39–41].

For consistency with other measures utilised within the IMPACT study, data collected using the MFQ was initially scored on a 4-point Likert scale ranging from 0 (“never”) to 3 (“always”; hereafter referred to as ‘alternate MFQ’). In the current study, depression severity was calculated using the original MFQ scoring procedure (hereafter referred to as ‘MFQ’ or ‘original MFQ’), with ratings of 2 and 3 combined. This was done in order to align with both the main trial paper [8] and other papers utilising this dataset [17, 42–44], and to avoid potential misinterpretation of the findings. The internal reliability in the current sample was excellent (α = 0.90).

Item 5 of the MFQ (‘I felt so tired I just sat around and did nothing’) was used as one of the primary measures for fatigue, recoded into a binary ‘fatigue status’ variable (0, 1 = ‘non-fatigued’; 2 = ‘fatigued’). Analyses using the alternate MFQ scoring procedure are available in supplementary materials.

The Health of the Nation Outcome Scale for Children and Adolescents (HoNOSCA) [45] is a 15-item scale used to measure overall current psychosocial impairment. All items are scored on a 5-point Likert scale ranging from 0 to 4, with 4 indicating highest severity. The final score is made up of the total score from the first 13 items. The HoNOSCA is well-validated and has demonstrated robust validity and reliability [45, 46]. The IMPACT study utilised the interview version of the HoNOSCA, which was adapted from the original clinical version. The internal reliability in the current sample was poor, bordering on questionable (α = 0.59).

### Data analysis

All variables were checked for normality and outliers using box plots, stem and leaf plots, and relevant tests. As the continuous data generally met the assumptions for parametric tests, and the intended analyses were robust to deviations from normality, this approach was used throughout.

Analyses were conducted in IBM SPSS Statistics (Version 29). As pre-registered, primary analyses were conducted on a complete case basis, with missingness reported; this is recommended when the proportion of missing data is large (i.e., over 40%) on important variables, as long as the limitations of the analysis are thoroughly discussed and hypothesis-generation is emphasised [47]. Two-tailed statistical tests are reported for all analyses, with the alpha set to 0.05. All primary analyses were conducted on both the MFQ and K-SADS fatigue items.

Prevalence of fatigue at baseline (RQ1) was assessed descriptively with count (*n*) and percentage (%) of participants meeting the criteria of ‘fatigued’. McNemar tests and Pearson’s correlation coefficients were employed to explore alignment between fatigue status as measured on the MFQ and K-SADS, and the appropriateness of a composite fatigue variable. Interpretations followed Cohen’s [48] conventions (small effect: *r* = .10; moderate effect: *r* = .30; large effect: *r* = .50).

To examine associations between baseline characteristics and fatigue status (RQ2), independent samples t-tests were used for continuous variables and binary logistic regressions for categorical variables. Binary logistic regressions were used to see if any variables uniquely predicted fatigue status over and above one another within a single model.

To assess the impact of treatment on binary fatigue status (RQ3), Generalized Estimating Equations (GEEs) were implemented using a normal distribution model with an unstructured correlation matrix, fitting time (baseline, 36-weeks, 86-weeks) as a within-subjects factor and arm (CBT, STPP, and BPI) as a between-subjects factor. Significant main or interactive effects for all analyses were resolved via pairwise comparisons.

Multiple linear regressions were run to assess whether baseline fatigue status predicted post-treatment improvement in depression symptom severity (RQ4). At step one, depression severity without MFQ item 5 at 36-weeks was entered as the dependent variable, with baseline depression severity, baseline fatigue status, medication status, and treatment arm entered as dummy-coded covariates (i.e., converted into three binary variables as opposed to one multi-categorical variable) to look at the main predictive effect across all treatments. At step two, the interaction terms of dummy-coded treatment arms multiplied by baseline fatigue status were entered to explore differential impact as a function of treatment arm. RQ5 took the same approach as RQ4, except depression severity was exchanged for psychosocial impairment.

## Results

### Prevalence of fatigue

At baseline, 340 (73.1%) participants were classified as fatigued on the MFQ, and 341 (73.8%) participants were classified as fatigued on the K-SADS. No significant difference was found between the proportion of individuals identified as fatigued between the MFQ and K-SADS (McNemar *p* = .868; see Table 1 for crosstabulation), yet the correlation between the two items was significant yet small (*r* = .18, *p* < .001), indicating that a composite fatigue variable would not be appropriate. Consequently, MFQ and K-SADS data are analysed separately going forward (see Sect.  2 of supplementary materials for analyses using the alternate MFQ).

**Table 1:** Crosstabulation of participants classified as fatigued on the original MFQ and K-SADS at baseline

MFQ	K-SADS		
	Fatigued (*n*)	Non-fatigued (*n*)	Total (*n*/*N*)
Fatigued	267	71	338
Non-fatigued	74	50	124
Total	341	121	462

Values are counts (*n*) of participants classified as fatigued vs. non-fatigued by each instrument at baseline (instrument-specific criteria). Row totals correspond to MFQ classifications; column totals to K-SADS classifications. *n* denotes participants with complete data on both measures; grand total *N* = 462

*K-SADS *Kiddie Schedule for Affective Disorders and Schizophrenia, *MFQ *Mood and Feelings Questionnaire

### Demographic and clinical characteristics of fatigue

Table 2 shows that adolescents who were classified as fatigued on the MFQ were significantly more likely to be older, an ethnic minority^1^, have more comorbid diagnoses, higher depression severity, and experience more psychosocial impairment at baseline (*p*’s < 0.03). However, there were no significant differences in sex or medication status (*p*’s > 0.116). Results from the K-SADS are displayed in Table 3 and were similar in relation to age and depression severity. However, there were no longer significant differences in ethnicity, comorbid diagnoses, or psychosocial impairment as a function of fatigue status at baseline (*p*’s > 0.083).

**Table 2:** Baseline characteristics of fatigued and non-fatigued participants on the original MFQ

Characteristic	Analysed	Fatigued	Non-fatigued	Comparison		
Continuous Variable	N	M (SD)	M (SD)	MD (95% CI)	*t*	*p*
Age	465	15.70 (1.41)	15.37 (1.44)	0.33 (0.04, 0.62)	2.21	027*
Comorbidities	371	1.00 (1.02)	0.70 (0.83)	0.27 (0.04, 0.50)	2.30	0.022*
Depression severity	465	47.78 (0.52)	40.22 (0.98)	7.56 (5.50, 9.61)	7.22	0.001*
Depression severity (adj.)	465	45.78 (0.52)	39.31 (0.97)	6.47 (4.41, 8.52)	6.18	0.001*
Psychosocial impairment	437	18.50 (0.35)	16.80 (0.47)	1.70 (0.42, 2.97)	2.61	0.009*

Categorical Variable	N	*n/N* (%)	*n/N* (%)	OR (95% CI)	Wald *χ²*	*p*
Female sex	465	261/340 (77%)	87/125 (70%)	1.44 (0.91, 2.28)	2.48	0.116
Minority ethnicity	450	57/326 (17%)	11/124 (9%)	2.18 (1.10, 4.31)	5.00	0.025*
On medication	456	62/332 (19%)	27/124 (22%)	1.21 (0.73, 2.02)	0.55	0.458

Continuous variables are presented as *M* (mean) and SD (standard deviation); categorical variables as *n/N* (%), where *n* = subgroup count and *N* = row-specific analysed sample with complete data. For continuous variables, MD (mean difference) with 95% CI (confidence interval) is tested with independent-samples *t* tests (*t*). For categorical variables, OR (odds ratio) with 95% CI is estimated via binary logistic regression with the Wald statistic (Wald). Comorbidities = number of co-occurring diagnoses (range 0–5). Depression severity (adj.) = score with the fatigue item (“I felt so tired I just sat around and did nothing”) removed. Coding: sex 0 = female, 1 = male; ethnicity 0 = ethnic minority, 1 = white; medication 0 = not taking, 1 = taking. Asterisks (*) denote *p* ≤ .05

**Table 3:** Baseline characteristics of fatigued and non-fatigued participants on the original K-SADS

Characteristic	Analysed	Fatigued	Non-fatigued	Comparison		
Continuous Variable	N	M (SD)	M (SD)	MD (95% CI)	*t*	*p*
Age	462	15.69 (0.08)	15.37 (0.13)	0.32 (0.03, 0.61)	2.13	0.033*
Comorbidities	368	0.94 (1.01)	0.74 (0.86)	0.20 (0.03, 0.43)	1.74	0.083
Depression severity	462	46.69 (10.44)	43.31 (10.21)	3.38 (1.22, 5.53)	3.07	0.002*
Depression severity (adj.)	462	44.93 (10.30)	41.75 (10.07)	3.18 (1.05, 5.31)	2.93	0.004*
Psychosocial impairment	434	18.36 (6.11)	17.30 (5.79)	1.07 (0.22, 2.36)	1.63	0.104

Categorical Variable	N	*n/N* (%)	*n/N* (%)	OR (95% CI)	Wald *χ²*	*p*
Female sex	462	261/340 (77%)	87/125 (70%)	1.41 (0.88, 2.23)	2.06	0.151
Minority ethnicity	447	57/326 (17%)	11/124 (9%)	0.85 (0.48, 1.50)	0.32	0.572
On medication	453	62/332 (19%)	27/124 (22%)	0.83 (0.48, 1.43)	0.45	0.502

Continuous variables are presented as *M* (mean) and SD (standard deviation); categorical variables as *n/N* (%), where *n* = subgroup count and *N* = row-specific analysed sample with complete data. For continuous variables, MD (mean difference) with 95% CI (confidence interval) is tested with independent-samples *t* tests (*t*). For categorical variables, OR (odds ratio) with 95% CI is estimated via binary logistic regression with the Wald statistic (Wald). Comorbidities = number of co-occurring diagnoses (range 0–5). Depression severity (adj.) = score with the fatigue item (“I felt so tired I just sat around and did nothing”) removed. Coding: sex 0 = female, 1 = male; ethnicity 0 = ethnic minority, 1 = white; medication 0 = not taking, 1 = taking. Asterisks (*) denote *p* ≤ .05

Additional regression analyses examined if any variables continued to uniquely predict fatigue status when combined into a single model. Depression severity with and without the fatigue item included were predictive of MFQ fatigue status (p’s < 0.001). However, only depression severity with the fatigue item included was predictive of K-SADS fatigue status (*p* = .045; see Table 4). See Sect. 3 of supplementary materials for analyses using the alternate MFQ.

**Table 4:** Predictors of fatigue status on the original MFQ and K-SADS

Characteristic	MFQ Comparison			K-SADS Comparison		
	OR (95% CI)	Wald	*p*	OR (95% CI)	Wald χ²	*p*
Age	1.10 (0.92, 1.32)	*1.08*	0.300	1.06 (0.89, 1.26)	0.38	0.540
Sex	0.91 (0.51, 1.64)	0.91	0.763	1.52 (0.87, 2.65)	2.13	0.144
Ethnicity	0.57 (0.25, 1.32)	1.72	0.190	0.97 (0.48, 1.97)	0.01	0.931
Medication	0.60 (0.21, 1.12)	2.37	0.124	1.13 (0.58, 2.21)	0.12	0.726
Comorbidities	1.26 (0.94, 1.69)	2.31	0.128	1.14 (0.87, 1.50)	0.91	0.340
Depression severity	1.07 (1.04, 1.10)	20.73	0.001*	1.03 (1.00, 1.05)	4.01	0.045*
Depression severity (adj.)	1.05 (1.03, 1.08)	14.41	0.001*	1.03 (1.00, 1.05)	3.68	0.055
Psychosocial impairment	1.04 (0.99, 1.09)	1.95	0.162	1.03 (0.98, 1.07)	1.15	0.285

OR (odds ratio) with 95% CI (confidence interval) is estimated via binary logistic regression with the Wald statistic (Wald). Comorbidities = number of co-occurring diagnoses (range 0–5). Depression severity (adj.) = score with the fatigue item removed. Coding: sex 0 = female, 1 = male; ethnicity 0 = ethnic minority, 1 = white; medication 0 = not taking, 1 = taking. Asterisks (*) denote *p* ≤ .05

### Changes in fatigue following treatment

Missingness across all timepoints is reported in Table 5 (see Sect.  7 of supplementary materials for further exploration of missingness). For MFQ fatigue status, GEEs found a significant main effect of time (OR = 2.23 [1.72, 2.90], Wald χ^2^ = 36.09, *p* < .001) but no significant time by arm interactions for CBT (*p* = .219) or STPP (*p* = .934) compared to BPI (active reference condition). McNemar test pairwise comparisons to resolve the main effect of time found significant reductions in fatigue status from baseline to post-treatment (*p* < .001) and further reductions post-treatment to follow-up (*p* = .045). Similar results were found on the K-SADS, with a significant main effect of time (OR = 3.01 [2.26, 4.01], Wald χ^2^ = 56.88, *p* < .001) but no time by arm interactions for CBT (*p* = .218) or STPP (*p* = .881) compared to BPI. Pairwise comparisons for the main effect of time also reported significant reductions in fatigue status from baseline to post-treatment (*p* < .001) and further reductions post-treatment to follow-up (*p* = .006). These findings indicate that treatment reduced clinically significant levels of fatigue amongst participants, and that this reduction continued across follow-up, irrespective of treatment arm. However, as indicated in Table 6, 31% on the MFQ and 23% on the K-SADS remained fatigued at follow-up. See Sect.  4 of supplementary materials for analyses using the alternate MFQ.

**Table 5:** Frequencies and percentages of available data on the original MFQ and KSADS at each timepoint

Timepoint	MFQ	K-SADS
	*n*/*N* (%)	*n*/*N* (%)
Baseline	465/465 (100%)	462/465 (99.35%)
Week 36	318/465 (68.39%)	278/465 (59.78%)
Week 86	352/465 (75.70%)	285/465 (61.29%)
Overall	282/465 (60.65%)	216/465 (46.45%)

Values are *n/N* (%), where *n* = number with non-missing data for that measure at the specified timepoint and *N* = 465 (analytic cohort). Percentages use *N* = 465 as the denominator at each timepoint for comparability. The Overall row indicates participants with complete data for that measure at all listed timepoints (Baseline, Week 36, Week 86)

*MFQ *Mood and Feelings Questionnaire, *K-SADS *Kiddie Schedule for Affective Disorders and Schizophrenia

**Table 6:** Frequencies and percentages of fatigued participants at each timepoint

Timepoint	CBT	STPP	BPI	Overall
	*n*/*N* (%)	*n*/*N* (%)	*n*/*N* (%)	*n*/*N* (%)
MFQ				
Baseline	118/154 (77%)	108/156 (69%)	114/155 (74%)	340/465 (73%)
Post-treatment	33/103 (32%)	37/109 (34%)	52/105 (50%)	122/318 (38%)
Follow-up	35/122 (29%)	34/114 (30%)	39/113 (35%)	108/352 (31%)
K-SADS				
Baseline	113/152 (74%)	111/156 (71%)	117/154 (76%)	341/462 (74%)
Post-treatment	25/87 (29%)	34/97 (35%)	36/94 (38%)	95/278 (34%)
Follow-up	17/94 (18%)	20/91 (22%)	27/100 (27%)	64/285 (23%)

Values are *n/N* (%), where *n* = fatigued and *N* = available cases for that measure within each arm and timepoint (percentages are within arm/timepoint). “Overall” pools across arms using the total *N* at that timepoint for each measure. Fatigue status is defined by instrument-specific criteria

*MFQ *Mood and Feelings Questionnaire, *K-SADS *Kiddie Schedule for Affective Disorders and Schizophrenia, *CBT *cognitive-behavioural therapy, *STPP *short-term psychoanalytic psychotherapy, *BPI *brief psychosocial intervention

### Baseline fatigue as a predictor of post-treatment depression symptom severity

Baseline MFQ fatigue status did not significantly predict levels of depression severity at 36-weeks (*p* = .716), which did not change when dummy-coded interaction terms between baseline fatigue status and treatment arms were added (*p*’s > 0.082). Similarly, baseline K-SADS fatigue status did not significantly predict depression severity at 36-weeks (*p* = .131), with interaction terms remaining non-significant (*p*’s > 0.069). See Sect.  5 of supplementary materials for analyses using the alternate MFQ.

### Baseline fatigue as a predicator of post-treatment psychosocial functioning

MFQ fatigue status at baseline did not significantly predict psychosocial functioning at 36-weeks (*p* = .683), with non-significant interaction terms between baseline fatigue status and treatment arms (*p*’s > 0.395). Results on the K-SADS was similarly non-significant (*p* = .259), with non-significant interaction terms (*p*’s > 0.500). See Sect.  6 of supplementary materials for analyses using the alternate MFQ.

## Discussion

This exploratory secondary analysis found that almost three-quarters of a large sample of clinically depressed adolescents were identified as having significant levels of fatigue, with similar prevalence between the self-report and diagnostic interview, yet with small correlations. Our primary analyses found that adolescents experiencing clinically significant fatigue were more likely to be older and an ethnic minority, with more comorbid diagnoses, higher depression severity, and higher levels of psychosocial impairment. However, these findings changed depending on whether fatigue was measured via self-report or diagnostic interview, and what the cut-off points for being classified as fatigued were. Subsequently, only age and depression severity were consistently associated across analyses of the original MFQ, alternate MFQ, and K-SADS. Overall, fatigue status decreased after receiving psychological treatments for depression, with no significant differences observed between CBT, STPP, or BPI, suggesting they all had an approximately equivalent impact. In this trial, experiencing fatigue at baseline did not significantly predict levels of depression severity or psychosocial functioning following treatment. Due to the exploratory nature of the study, these findings should be considered as tentative.

Fatigue is a common symptom of adolescent depression. The rate of fatigue in our sample^2^ falls in between other reports across the wider literature (e.g., 43% [9]; 97.3% [10]), which is potentially reflective of differences in the samples and measures used. As the current study has a much larger sample size of clinically referred adolescents, it is more likely to be precise. Furthermore, fatigue was consistently found to be associated with older age and higher depression severity, which reflects previous research on fatigued adolescents in community and primary care samples [49, 50] and provides an indication of certain characteristics to look out for during assessment. This, in turn, may alter treatment approaches and how depression is managed. On the original MFQ only, fatigue was also associated with being an ethnic minority, more comorbid diagnoses, and experiencing higher psychosocial impairment; however, these characteristics were not significantly associated with fatigue when measured by the K-SADS.

Interestingly, although prevalence and demographic patterns demonstrated some similarities across measures, the correlation between the MFQ and K-SADS fatigue items was small, suggesting they are not consistently identifying the same individuals. One possible explanation for this limited convergence is the distinction between interviewer-rated and self-reported measures, which may capture different aspects of fatigue. As qualitative research has highlighted the subjectivity of fatigue, including its multifaceted, dynamic nature, this is perhaps unsurprising [2, 51, 52]. The wording between the two measures is also different, with the MFQ focusing on tiredness and subsequent lack of activity, and the K-SADS focusing on somatisation and the frequency and duration of fatigue. Further, the K-SADS provides interviewers with prompts to aid the identification of fatigue, whereas the MFQ does not. This explanation is supported by regression analyses showing that K-SADS fatigue was only associated with depression severity when the fatigue item was included, whereas MFQ fatigue remained associated even when fatigue was removed from the total score. When fatigue items were included in both measures, associations with depression severity aligned more closely, indicating some shared variance at the symptom level. Taken together, this pattern tentatively suggests that differences in measurement approach may contribute to this discrepancy; however, this interpretation is speculative and requires further empirical testing.

Encouragingly, the current study found that fatigue significantly decreased from baseline to post-treatment to long-term follow-up across all three treatment arms. Further, the presence of fatigue at baseline did not significantly predict how well these treatments addressed depression severity or psychosocial functioning, despite significant associations between these characteristics and fatigue at baseline. This indicates that current psychological treatments for depression utilised in routine clinical practice do seem to reduce the presence of fatigue, and that fatigue does not impact the efficacy of treatments. However, this could be because some clinicians within the IMPACT trial were already adjusting their approaches to better accommodate fatigue; for example, in Herring et al. [31], which used data from IMPACT-My Experience (the longitudinal qualitative study embedded within the IMPACT trial) to explore the experiences of CBT in depressed adolescents reporting fatigue, some participants described how sessions lengths were shortened to be less tiring, or were scheduled less frequently so that rest days were not interrupted. Additionally, these findings could reflect that treatments utilised within clinical practice already incorporate techniques present in interventions designed to target fatigue [53]; therefore, fatigue may already be implicitly addressed during psychological treatments for adolescent MDD. Further research is needed to confirm this.

However, whilst reductions in fatigue across treatments were statistically significant, 23% to 31% of adolescents in the current study still met the criteria for clinically significant fatigue at 86-weeks follow-up. These statistics are somewhat similar to reports in the adult literature regarding fatigue as a residual symptom of MDD (e.g., 35% [19]; 23% [20]), and align with a recent network analysis of the IMPACT data, which identified fatigue as having strong autocorrelations over time and a tendency to persist regardless of treatment [17]. This indicates that whilst these interventions seem effective at face value in reducing fatigue severity and status in depressed adolescents, they do not work for everyone. There are many potential reasons why fatigue may persist following psychological treatment for MDD, including neurobiological dysregulation, comorbidities, behavioural maintenance factors, and fatigue as an independent symptom rather than a by-product of depression [54, 55]. It may be that further explicit adaptations or augmentations are needed to ensure that psychological treatments for adolescent depression are better able to target fatigue, such as modules or booster sessions targeting specific domains of behaviour change [53]. However, further research is needed to develop and test the effectiveness of such an approach.

### Strengths and limitations

The data used within this post-hoc exploratory secondary analysis was from a high-quality RCT with a large sample of adolescents clinically diagnosed with MDD through a gold standard diagnostic interview. This increases the diagnostic reliability of the sample, and the robustness of the findings. Participants were recruited from 15 clinics across three regions of the UK, comprising both rural and urban populations, which increases the generalisability of the sample to the wider UK adolescent population. Further, participants were followed up over an 86-week period, meaning the long-term effects of the treatments could be assessed; this is particularly important in the context of MDD and rates of relapse [56]. Our data analysis plan was also pre-registered, which aids the transparency, reliability, and replicability of the research.

However, there are some limitations which must be considered when interpreting the findings. Firstly, a complete case analysis was conducted, which was appropriate for the aims of the study and simplicity, alongside the high percentage of missing data on key variables, and analyses which indicated that data may be Missing Not at Random (MNAR) [47]; see supplementary materials for missingness analyses). However, the complete case analysis does mean that findings may be vulnerable to bias (e.g., were the participants lost to follow-up more likely to be fatigued? ) and result in a loss of statistical power [57]. This emphasises the importance of the study being hypothesis-generating rather than hypothesis-confirming. Further, fatigue was measured using single items extracted from larger measures of depression. Although this approach was necessary as a specific measure of fatigue was not included in the IMPACT trial, fatigue is a multidimensional construct which cannot be adequately captured by a singular item [33, 51]. It could be that more participants experienced fatigue but did not report it because the single items did not represent the different facets of their experience (i.e., mental fatigue, physical fatigue, severity, impairment, etc.). These single items also included a limited range, which could have impacted the chances of getting strong correlations and may have contributed to the null findings. Additionally, the Cronbach’s alpha for the HoNOSCA was poor, indicating a lack of reliability; the findings regarding psychosocial impairment should be interpreted with this in mind. Further, whilst findings were largely consistent, some results did vary with secondary analyses, indicating that some findings (e.g., fatigue severity decreasing with treatment) are more robust than others (e.g., associations between fatigue and sex). However, it is important to recognise that, due to the exploratory nature of this study, multiple tests were run, which increases the likelihood of Type I errors (i.e., false positives); as such, the findings must be considered as tentative, with further research required. Finally, power calculations were not conducted for the current secondary analysis, making it important to interpret some the findings (i.e., predictive analyses) with caution.

## Conclusion

This secondary analysis of data from a large-scale RCT highlights that fatigue is commonly reported by depressed adolescents, particularly those who are older and experiencing higher depression severity. However, psychological treatments for depression, such as CBT, STPP and BPI, all seem to reduce fatigue, and the presence of fatigue does not seem to predict how well these treatments work for depression or psychosocial functioning. Despite this, up to one third of depressed adolescents still reported residual fatigue following treatment. As a post-hoc secondary analysis intended for hypothesis generation, these findings indicate a need for further research, specifically focused on incorporating multidimensional fatigue measures within large-scale RCTs of psychological treatments for adolescent depression, and determining whether adaptations to treatments can address issues with residual fatigue.

## Supplementary Information

Supplementary Material 1.

## Data Availability

Research data were obtained from the IMPACT study Principal Investigators. Requests for data should be directed to Professor Ian Goodyer.

## Footnotes

1 As most of the sample identified as white (82.2%), a binary ethnicity variable was created where a score of 0 indicated ‘ethnic minority’, which encompassed Mixed, Asian/Asian British, Black/Black British, and Other ethnicities, and a score of 1 indicated ‘white’.

2 In the current study, 73.8% of adolescents reported fatigue on the K-SADS. This statistic differs from the 73.3% reported by Goodyer et al. (2017), as the main trial outcomes paper followed intention-to-treat analyses, whereas we conducted complete case analyses.
