# Supplementary material for: Exploring fatigue in clinically depressed adolescents: a secondary analysis of the IMPACT study sample
Source: BMC Psychol. 2026 Jun 12;14:1208. doi: 10.1186/s40359-026-04942-3 (PMC13483419; doi:10.1186/s40359-026-04942-3)
Supplement: Supplementary file 1 [file 40359_2026_4942_MOESM1_ESM.docx]

Supplementary materials

This document contains additional analyses using alternate scoring for the Mood and Feelings Questionnaire (MFQ), which was used during IMPACT study data collection but does not reflect how the MFQ is commonly reported (sections 1-6). This document also includes analyses regarding attrition and missingness (section 7).

**1. Alternate MFQ scoring**

Item 5 of the MFQ (‘I felt so tired I just sat around and did nothing’), using the IMPACT scoring procedure (4-point Likert scale ranging from 0/“never” to 3/“always”; hereafter referred to as ‘alternate MFQ’), was used as a secondary measure for fatigue. This was in order to address issues regarding sensitivity and limited range when conducting analyses based on a single item.

**2. Prevalence of fatigue**

Baseline fatigue data was available for 100% (*n* = 465) of participants on the alternate MFQ, with 25.6% (*n* = 119) of the sample meeting the threshold for clinically significant fatigue. This is in comparison to 73.1% (*n* = 340) on the original MFQ.

The McNemar test found significant differences between the alternate MFQ and K-SADS (*p* < .001), with only 99 participants identified as fatigued on both items. Pearson’s correlations indicated a positive association but small effect between the alternate MFQ and the K-SADS at baseline (*r* = .18, *p* < .001), further reinforcing the conclusion from the primary analysis that a composite fatigue variable would be inappropriate.

**3. Demographic and clinical characteristics of fatigue**

Table S1 summarises baseline characteristics of participants with or without fatigue on the alternate MFQ, with continuous variables analysed using individual samples t-test and categorical variables analysed with binary logistic regression. The only identified difference when compared to the primary analyses on the original MFQ was that adolescents classified as fatigued on the alternate MFQ were significantly more likely to be female (*p* = .008).

Table S1. Baseline characteristics of fatigued and non-fatigued participants on the alternate MFQ.

| Characteristic | Analysed | Fatigued | Non-fatigued | Comparison |  |  |
| --- | --- | --- | --- | --- | --- | --- |
| Continuous Variable | N | M (SD) | M (SD) | MD (95% CI) | *t* | *p* |
| Age | 465 | 15.83 (1.39) | 15.53 (1.43) | 0.30 (0.01, 0.60) | 2.01 | .008* |
| Comorbidities | 371 | 1.08 (1.14) | 0.83 (0.92) | 0.26 (0.03, 0.49) | 2.22 | .027* |
| Depression severity | 465 | 51.41 (8.67) | 43.90 (10.44) | 7.61 (5.52, 9.70) | 7.15 | .001* |
| Depression severity (adj.) | 465 | 49.41 (8.67) | 42.19 (10.31) | 7.22 (5.15, 9.29) | 6.85 | .001* |
| Psychosocial impairment | 437 | 19.50 (6.85) | 17.54 (5.64) | 1.95 (0.69, 3.24) | 2.99 | .003* |
| Categorical Variable | N | *n/N* (%) | *n/N* (%) | OR (95% CI) | Wald *χ²* | *p* |
| Female sex | 465 | 100/119 (84%) | 248/346 (72%) | 2.08 (1.21, 3.58) | 2.48 | .008* |
| Minority ethnicity | 450 | 26/85 (31%) | 42/339 (12%) | 2.16 (1.25, 3.73) | 5.00 | .006* |
| On medication | 456 | 26/116 (22%) | 63/340 (19%) | 0.79 (0.47, 1.32) | 0.55 | .363 |

Note. Continuous variables are presented as *M* (mean) and SD (standard deviation); categorical variables as *n/N* (%), where *n* = subgroup count and *N* = row-specific analysed sample with complete data. For continuous variables, MD (mean difference) with 95% CI (confidence interval) is tested with independent-samples *t* tests (*t*). For categorical variables, OR (odds ratio) with 95% CI is estimated via binary logistic regression with the Wald statistic (Wald). Comorbidities = number of co-occurring diagnoses (range 0–5). Depression severity (adj.) = score with the fatigue item (“I felt so tired I just sat around and did nothing”) removed. Coding: sex 0 = female, 1 = male; ethnicity 0 = ethnic minority, 1 = white; medication 0 = not taking, 1 = taking. Asterisks (*) denote *p* ≤ .05.

Table S2 reports the findings from the regression analysis exploring whether any demographic and clinical variables predicted fatigue status over and above one another when combined into a single model. Similar to the primary analysis, only depression severity with and without the fatigue item included were significant predictors of fatigue status at baseline on the alternate MFQ (*p*’s < .001).

Table S2. Predictors of fatigue status on the alternative MFQ.

| Characteristic | OR (95% CI) | Wald *χ²* | *p* |
| --- | --- | --- | --- |
| Age | 1.07 (0.87, 1.30) | 0.38 | *p* = .537 |
| Sex | 0.83 (0.43, 1.60) | 0.32 | *p* = .573 |
| Ethnicity | 0.53 (0.27, 1.07) | 3.13 | *p* = .077 |
| Medication | 1.21 (0.61, 2.38) | 0.29 | *p* = .588 |
| Comorbidities | 1.10 (0.85, 1.43) | 0.56 | *p* = .456 |
| Depression severity | 1.10 (1.06, 1.14) | 27.11 | *p* < .001* |
| Depression severity (adj.) | 1.10 (1.06, 1.14) | 25.06 | *p* < .001* |
| Psychosocial impairment | 1.02 (0.97, 1.07) | 0.59 | *p* = .441 |

Note. OR (odds ratio) with 95% CI (confidence interval) is estimated via binary logistic regression with the Wald statistic (Wald). Comorbidities = number of co-occurring diagnoses (range 0–5). Depression severity (adj.) = score with the fatigue item removed. Coding: sex 0 = female, 1 = male; ethnicity 0 = ethnic minority, 1 = white; medication 0 = not taking, 1 = taking. Asterisks (*) denote *p* ≤ .05.

**4. Changes in fatigue following treatment**

Table S3 reports the number of individuals with clinically significant fatigue on the alternate MFQ at each time point.

Table S3. Frequencies and percentages of fatigued participants on the alternate MFQ at each timepoint.

| Timepoint | CBT | STPP | BPI | Overall |
| --- | --- | --- | --- | --- |
|  | n/N (%) | n/N (%) | n/N (%) | n/N (%) |
| Baseline | 36/154 (23%) | 36/156 (23%) | 47/155 (30%) | 119/465 (26%) |
| Post-treatment | 6/103 (6%) | 11/109 (10%) | 11/105 (11%) | 28/317 (9%) |
| Follow-up | 11/122 (9%) | 9/114 (8%) | 5/113 (4%) | 25/349 (7%) |

Note. CBT = cognitive-behavioural therapy; STPP = short-term psychoanalytic psychotherapy; BPI = brief psychosocial intervention. Values are *n/N* (%), where *n* = fatigued and *N* = available cases for that measure within each arm and timepoint (percentages are within arm/timepoint). “Overall” pools across arms using the total *N* at that timepoint for each measure. Fatigue status is defined by instrument-specific criteria.

GEEs for clinically significant fatigue measured on the alternate MFQ found similar results to the primary analyses, with a significant main effect of time (OR = 3.24 [2.18, 4.82], Wald χ^2^ = 33.54, *p* < .001) but no significant time by arm interactions for CBT (*p* = .114) or STPP (*p* = .619) compared to BPI. Unlike the primary analyses, McNemar test pairwise comparisons to resolve the main effect of time found significant changes in fatigue from baseline to post-treatment (*p* < .001), but not from post-treatment to follow-up (*p* = .500).

**5. Baseline fatigue as a predictor of post-treatment depression symptom severity**

Similar to the primary analyses, clinically significant fatigue status at baseline as measured on the alternate MFQ did not significantly predict depression severity at 36-weeks (*p* = .627)^[1]^, with non-significant interaction terms (*p*’s > .468).

**6. Baseline fatigue as a predictor of post-treatment psychosocial functioning**

Clinically significant fatigue status at baseline on the alternate MFQ did not significantly predict levels of psychosocial functioning at 36-weeks (*p* = .855), with non-significant interaction terms (*p*’s > .293).

**7. Missingness**

Attrition rates were notably high, ranging from 0 to 44.1% across variables. Table S4 summarises the frequencies and rates of missingness across key variables.

Table S4. Frequencies and percentages of fatigued participants on the alternate MFQ at each timepoint.

| Variable | Reported | Missing |
| --- | --- | --- |
|  | n/N (%) | n/N (%) |
| Treatment Group | 465/465 (100%) | 0/465 (0%) |
| Age | 465/465 (100%) | 0/465 (0%) |
| Sex | 465/465 (100%) | 0/465 (0%) |
| Ethnicity | 465/465 (100%) | 0/465 (0%) |
| MFQ (baseline) | 465/465 (100%) | 0/465 (0%) |
| MFQ (Week 36) | 318/465 (68%) | 147/465 (32%) |
| MFQ (Week 86) | 352/465 (76%) | 113/465 (24%) |
| HoNOSCA (baseline) | 437/465 (94%) | 28/465 (6%) |
| HoNOSCA (Week 36) | 260/465 (56%) | 205/465 (44%) |
| HoNOSCA (Week 86) | 274/465 (59%) | 191/465 (41%) |
| K-SADS (baseline) | 462/465 (99%) | 3/465 (1%) |
| K-SADS (Week 36) | 278/465 (60%) | 187/465 (40%) |
| K-SADS (Week 86) | 285/465 (61%) | 180/465 (39%) |

To assess whether the missing data could be considered Missing Completely At Random (MCAR), Little's MCAR test was applied. The results indicated that the data was not MCAR, χ²(176) = 238.99, *p* = .001. Hence, the pattern of missingness is not purely random and may be related to observed or unobserved variables.

Given this, a series of logistic regression analyses were conducted to determine whether the data was Missing at Random (MAR) or Missing Not at Random (MNAR). For variables with more than 5% missingness, a binary indicator of missingness (1 = missing, 0 = not missing) was used as the dependent variable. The covariates included arm, age, sex, ethnicity, MFQ total at each timepoint (i.e., baseline, 36-weeks, 86-weeks), HoNOSCA total at each timepoint (i.e., baseline, 36-weeks, 86-weeks), and K-SADS total at each timepoint (i.e., baseline, 36-weeks, 86-weeks).

The logistic regression analyses identified a significant model, several significant predictors of missingness, and a substantial *R*^2^ for HoNOSCA at baseline, suggesting that missingness in this variable may be related to the observed data, and thus MAR. However, the non-significant models and low *R*^2^ for other missingness variables indicate that their missingness may be MNAR. Table S5 summarizes the significant predictors for each missingness variable.

Table S5. Summary of significant predictors.

| Missingness Variable | Significant predictors | *χ*^2^ | *p* | Nagelkerke *R*^2^ |
| --- | --- | --- | --- | --- |
| MFQ (Week 36) | N/A | 7.06 | .795 | .05 |
| MFQ (Week 86) | N/A | 14.32 | .216 | .13 |
| HoNOSCA (Week 86) | MFQ (36-weeks), HoNOSCA (36-weeks) | 20.63 | .037 | .42 |
| HoNOSCA (Week 36) | HoNOSCA (baseline) | 10.53 | .484 | .06 |
| HoNOSCA (Week 86) | N/A | 14.13 | .226 | .09 |
| K-SADS (Week 36) | N/A | 5.04 | .929 | .03 |
| K-SADS (Week 86) | N/A | 13.75 | .247 | .09 |

Note. Variables of age, sex, ethnicity, MFQ at baseline, and K-SADS at baseline had missingness at less than 5% and thus were not included as the dependent variable.

1. P values are from step 2 of the regression model; where there are multiple values, the lowest is reported.
